# Increased level of TXNIP and nuclear translocation of TXN is associated with end stage renal disease and development of multiplex renal tumours

**DOI:** 10.1186/s12882-024-03653-4

**Published:** 2024-07-17

**Authors:** Tamas Beothe, Janos Docs, Gyula Kovacs, Lehel Peterfi

**Affiliations:** 1 Department of Urology, Peterfy Sandor Hospital, Budapest, Hungary; 2 Department of Urology, Medical School, University of Pecs, Munkacsy Mihaly u. 2, Pecs, 7621 Hungary; 3 Medical Faculty, Ruprecht-Karls-University, Heidelberg, Germany

**Keywords:** End stage kidney, TXNIP, TXN, Cancer, Immunohistochemistry

## Abstract

**Background:**

End-stage and acquired cystic renal disease (ESRD/ACRD) kidneys are characterized by inflammatory remodelling and multiplex renal cell carcinomas (RCC). Eosinophilic vacuolated tumour (EVT) occurs exclusively in ACRD. The aim of this study was to identify the involvement of thioredoxin-interacting protein (TXNIP) and thioredoxin (TXN) in ESRD/ACRD pathology.

**Methods:**

Expression of TXNIP and TXN was examined in histological slides of 6 ESRD and 6 ACRD kidneys, precursor lesions and associated tumours as well as of RCCs from the general population by immunohistochemistry.

**Results:**

Strong TXNIP expression was seen in epithelial cells, myo-fibroblasts and endothelial cells and weak TXN expression in ESRD/ACRD kidneys and tumours. In ACRD specific EVT and its precursors TXN were translocated into nuclei.

**Conclusion:**

The impaired TXNIP/TXN redox homeostasis might be associated with development of multiplex cancer especially of EVT in ESRD/ACRD kidney.

## Background

End-stage renal disease (ESRD) is the final stage of distinct types of kidney disease resulting in loss of renal function. Due to increasing number of diabetes mellitus type 2 in the population diabetic nephropathy (DN) is now the leading cause of ESRD [1]. Due to continuing metabolic stress, hyperglycaemia and increased level of ROS tubular cells express pro-inflammatory, pro-fibrotic cytokines resulting in excessive production of extracellular matrix [2]. Normal kidney structure in ESRD is replaced by inflammatory fibrotic tissue and long-term haemodialysis results in so called “acquired cystic renal disease” (ACRD) [3].

Renal cell tumour develops in ESRD/ACRD kidney more frequently than in the general population [4–6]. Conventional, papillary and chromophobe RCCs in ESRD/ACRD kidneys display morphologies and genetics like those of the general populations [7, 8]. The so-called eosinophilic vacuolated tumour (EVT) with unusual histology and genetics occurs preferentially in ACRD kidneys [9]. A strong similarity has been shown between wound healing and progressive inflammatory fibrosis in ESRD/ACRD, but molecular basis of tumour development in ESRD/ACRD kidneys is not yet known [6]. IL-6 seems to be the main driver of progressive inflammation and kidney remodelling [10]. IL6 triggers the production of reactive oxygen species (ROS) which may lead to mitochondrial and genomic DNA damage and to development of pre-neoplastic lesions and cancer. [11, 12].

Thioredoxin interacting protein (TXNIP), a member of the alpha arrestin protein family, controls the intracellular ROS production through inhibiting the anti-oxidative function of thioredoxin (TXN). The imbalance of TXNIP and TXN expression results in accumulation of ROS and increased cellular stress. Elevated TXNIP expression and oxidative stress influence several biological functions, regulates cell growth, differentiation, and apoptosis. The oxidative and nutritional stress results in damaging the proximal tubular cells which release proinflammatory cytokines. TXNIP is an important regulator of endothelial cell survival and angiogenesis and play a crucial role in the vascular endothelial cell dysfunction and angiogenesis in diabetic nephropathy [13, 14]. This study aimed to determine of TXNIP and TXN expression and their cellular localisation in ESRD/ACRD kidneys, tumour precursor lesions and tumours by immunohistochemistry.

## Materials and methods

### Kidney tissue and tumour specimen

Six ESRD and six ACRD kidneys removed due to cancer in distinct European countries between 1995 and 1998 were selected for this study (see Acknowledgements). Each kidney was worked up entirely and processed in paraffin blocks for histological analysis. The haematoxylin and eosin-stained slides were scored for cysts, small precursor lesions and tumours as described previously [10]. The diagnosis of the main tumours was established according to the Heidelberg Classification [15]. All together, we diagnosed five papillary RCC (pRCC), six conventional RCC (cRCC), one renal oncocytoma, three ACRD-associated, eosinophilic-vacuolated tumour (EVT), four chromophobe RCC (chRCC), and one clear cell pRCC in the 12 kidneys. We have included three EVT obtained from other institutes for consultation, two of them contained oxalate crystals within the tumour tissue. We also counted 65 small papillary precursor lesions, some of them with psammoma bodies, 40 chromophobe-like and 24 eosinophilic vacuolated pre-neoplastic lesions of microscopic size in the 12 ESRD/ACRD kidneys. In addition, 36 proliferative cysts, including 10 with solid-papillary growth of large eosinophilic vacuolated cells were counted [16]. Tissue array containing three 0.6 mm core biopsies each of the main tumours, cRCCs and pRCCs from the general population were made by using the Manual Tissue Arrayer (MTA1, Beecher Instruments Inc., Sun Prairie, USA). Tissue arrays of cRCCs [17] and pRCC were constructed from tumours of the general population operated between 2000 and 2015 at the Department of Urology, University of Pecs, Hungary.

### Immunohistochemistry

Sixteen representative paraffin blocks from ESRD/ACRD kidneys, three of them from other institutes, and for control paraffin blocks from three normal adult kidneys obtained by tumour nephrectomy and TMAs were selected for immunohistochemistry. TMAs of cRCCs were stained previously with TXNIP antibody [17] and with TXN for this study. TMA of pRCCs was stained with both TXN and TXNIP for this study. After removing the paraffin and rehydration the 4 μm sections were subjected to heat-induced epitope retrieval in citrate buffer, pH 6.0 for TXN and EnVision FLEX Target Retrieval Solution, high pH (DAKO, Glostrup, Denmark) for TXNIP in 2100-Retriever (Pick-Cell Laboratories, Amsterdam, The Netherlands). Endogenous peroxidase activity was blocked with Envision FLEX Peroxydase Blocking Reagent (DAKO) for 10 min at room temperature. One series of the consecutive slides were then incubated for one hour with monoclonal rabbit anti-TXNIP antibody (EPR14774, ab 188,865, abcam, Cambridge, UK) at the dilution of 1:200 and the second series with polyclonal rabbit anti-TXN antibody (HPA055752, Atlas Antibodies AB, Bromma, Sweden) at the dilution of 1:500, at room temperature. EnVision FLEX horse-radish-peroxidase conjugated secondary antibody (DAKO) was applied for 30 min at room temperature and colour was developed using the DAB substrate (DAKO). Tissue sections were counterstained with Mayer’s haematoxylin (Lillie’s modification, DAKO) and after 10 s bluing mounted by Pertex. For negative control the primary antibody was omitted and for positive control we used the normal kidney biopsies.

## Results

### Expression of TXN and TXNIP in end stage kidneys

In normal adult kidneys TXNIP was expressed in the cytoplasm of distal tubular cells and stromal endothelial cells, whereas TXN displayed a weak expression in proximal tubular cells. However, after strong inflammatory remodelling the origin of tubular components of the end stage kidney, even after staining with tubular markers, remained uncertain.

**Fig. 1:**
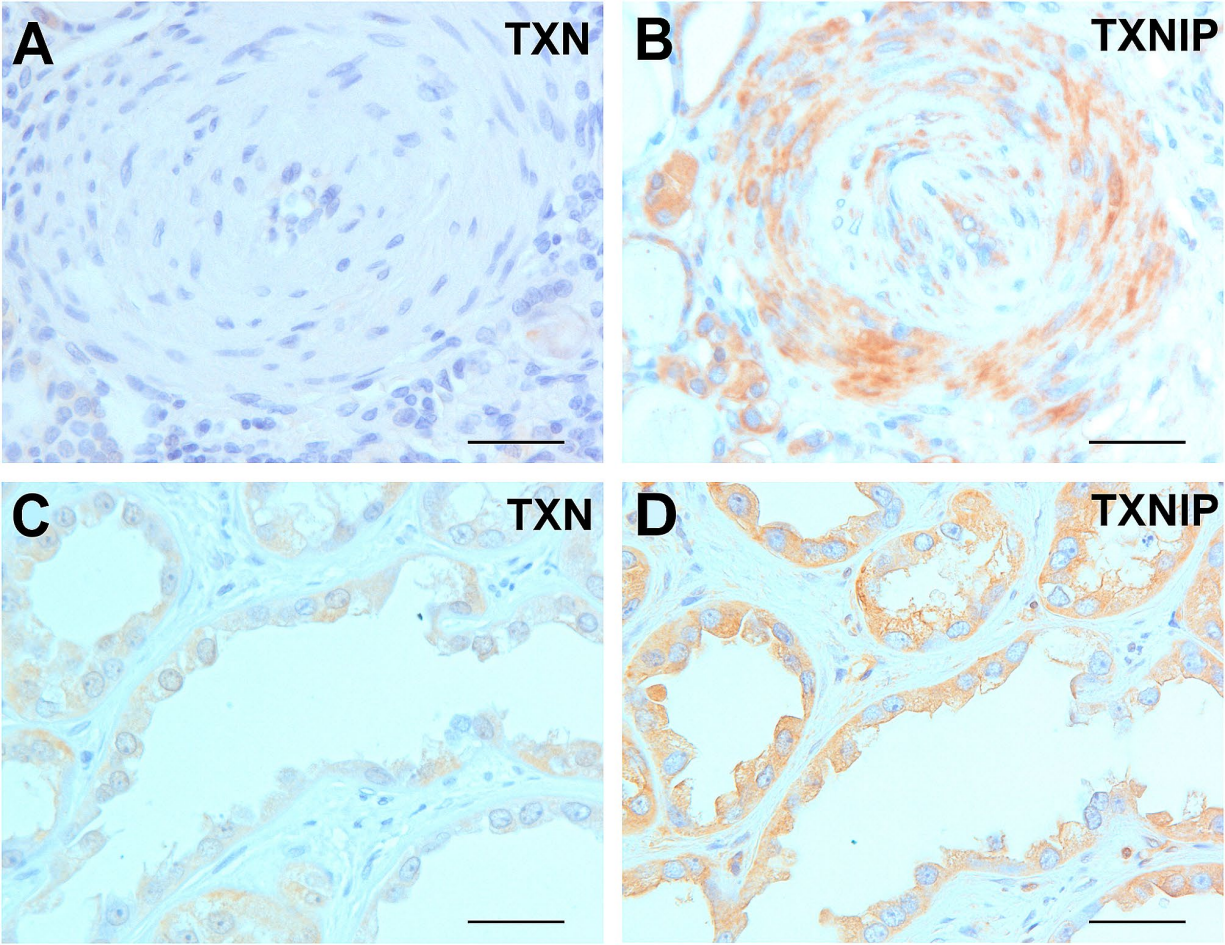
Expression of TXN and TXNIP in non-tumorous end-stage kidneys. (**A)** Lack of expression of TNX in thick arterial wall and endothelial cells. (**B)** Expression of TXNIP in myofibroblast and endothelial cells. (**C)** Weak expression of TXN in cells of dilated tubules. No nuclear translocation of TXN protein. (**D)** Strong cytoplasmic and membranous expression of TXNIP in tubules in the same area of the kidney. Scale bars = 50 μm

In ESRD/ACRD kidneys strong TXNIP expression was seen in proliferating myofibroblast in arterial wall and in endothelial cells. Several medium sized or smaller blood vessels displayed arterial wall thickening and were obliterated by TXNIP positive endothelial cells. The pericytes and myofibroblasts showed strong positive staining with the TXNIP antibody, whereas the TXN immunoreaction was negative (Fig. 1A, B). Circumscribed proliferation of small vessels embedded in inflammatory stroma observed in all slides showed strong positive TXNIP staining. Strong positive TXNIP staining was seen in cell clusters without lumen and single epithelial cells embedded in hyalinized stroma but none of them showed nuclear positivity. Immunohistochemistry revealed weak cytoplasmic TXN reaction in epithelial cell groups and in small atrophic as well as in dilated tubules lined with flat or cuboidal epithelial cells. Dilated tubules and small cysts showed weak TXN and stronger TXNIP positive immunoreaction, but no nuclear staining (Fig. 1C, D).

### Expression of TXN and TXNIP in tumours and precursor lesions

**Fig. 2:**
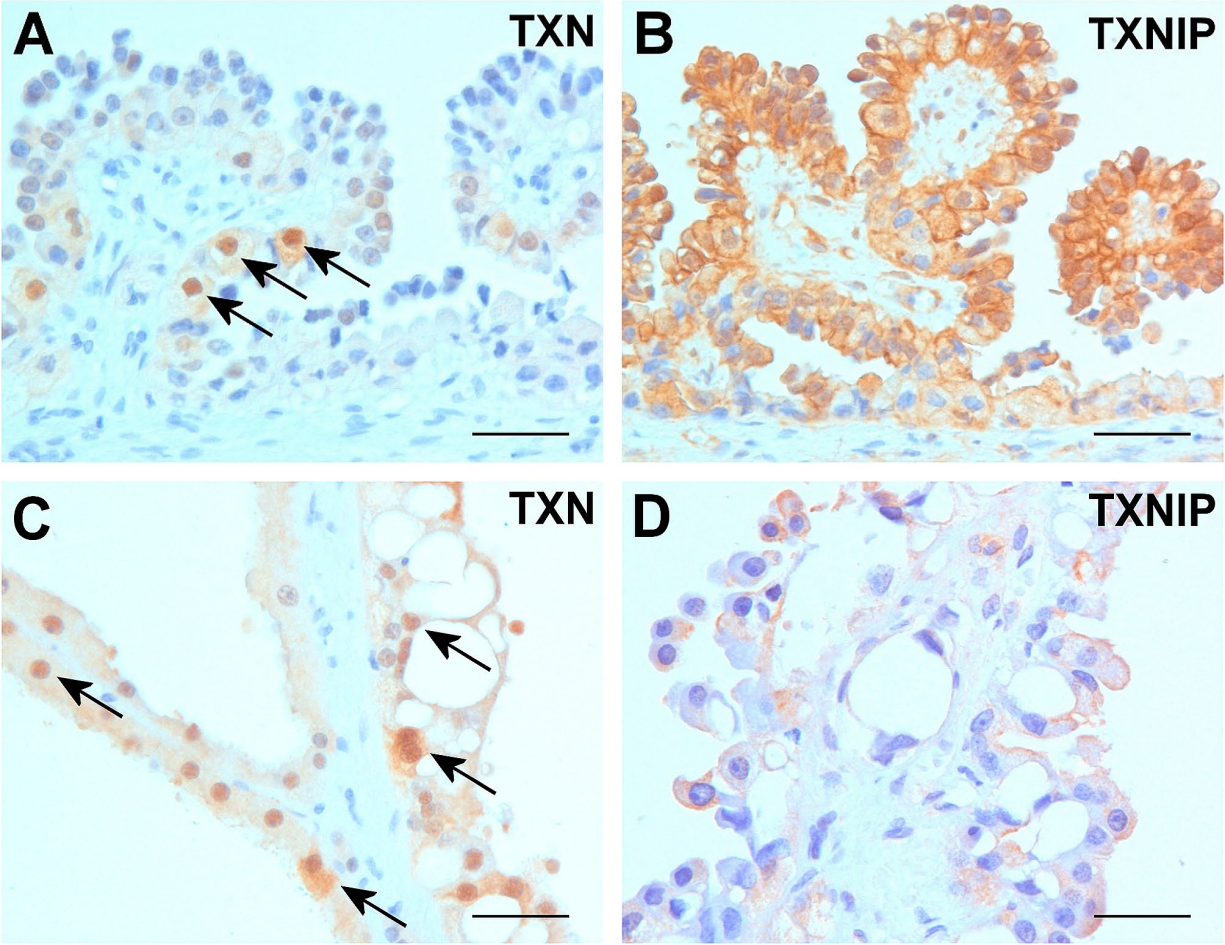
Expression of TXN and TXNIP in proliferative cysts. (**A**) Strong expression of TXN in nucleus of papillary growing structures (arrows). (**B)** The same papillary growing cells display a cytoplasmic and membranous TXNIP immunostaining. (**C)** Proliferative cysts lined with large vacuolated cells displaying nuclear TXN positivity (arrows). (**D)** Another proliferative cyst with vacuolated cells showing membranous TXNIP staining. Scale bars = 50 μm

In ACRD kidneys several smaller or larger proliferative cysts lined with papillary growing epithelial cells and 10 cysts with eosinophilic vacuolated cells were seen. Proliferating cysts lined by papillary growing epithelial cells displayed in approximately 20–30% of the cells weak cytoplasmic and a strong nuclear TNX positive staining (Fig. 2A). The same papillary growing intra-cystic epithelial cells displayed a strong cytoplasmic and membranous positivity with the TXNIP antibody (Fig. 2B). The large eosinophil vacuolated cysts showed weak TXN positive immune reaction in the cytoplasm and occasionally strong nuclear positivity (Fig. 2C). The large vacuolated cells displayed cytoplasmic and membranous TXNIP staining (Fig. 2D). Considering all results, the TXNIP showed higher level of expression in higher number of epithelial cells in ESRD/ACRD kidneys than as TXN. The eosinophil vacuolated cells in cystic precursor lesions and proliferative cysts showed nuclear translocation of TXN.

**Fig. 3:**
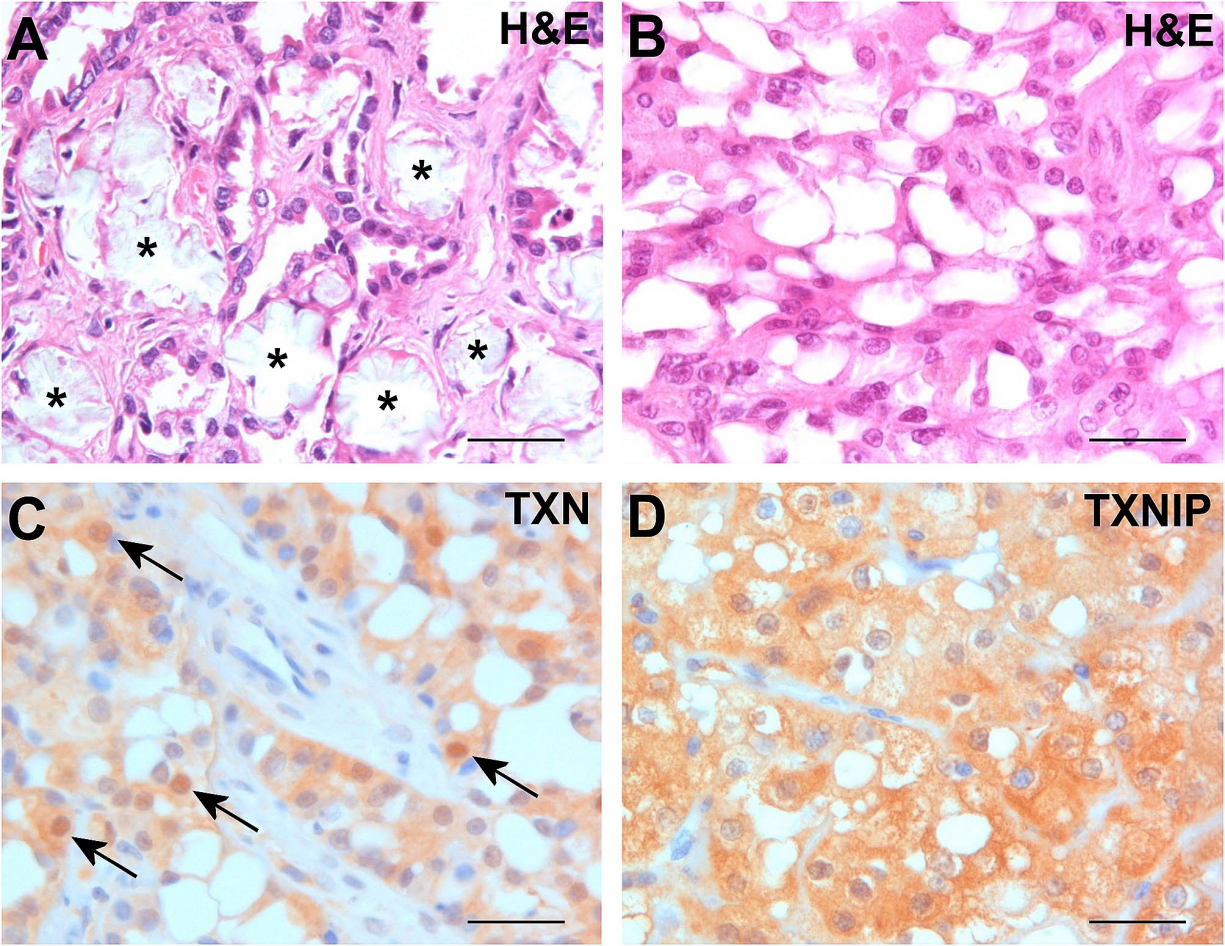
Eosinophil vacuolated tumour. (**A)** Several oxalate crystals (stars) disrupting the tumorous tissue. (**B)** Eosinophil vacuolated tumour. (**C)** Cytoplasmic and nuclear staining (arrows) of EVT cells with TXN. (**D)** Cytoplasmic and membranous positivity of EVT with TXNIP antibody. Scale bars = 50 μm

Two of the 6 EVT displayed oxalate crystals, others showed typical eosinophilic vacuolated cytoplasm (Fig. 3A B). The six EVT showed variable intensity of cytoplasmic TXN staining and 5 displayed a nuclear positivity as well (Fig. 3C). Strong cytoplasmic and membrane positivity and occasionally nuclear staining was seen with TXNIP antibody in cells of EVT (Fig. 3. D). The four chRCCs displayed cytoplasmic TXN and TXNIP staining and 2 and 1 nuclear staining, respectively. Each of the 11 papillary pre-neoplastic lesions in original slides as well as the five pRCCs included into the TMA showed cytoplasmic positive staining with TXNIP antibody. Medium level of TXN staining was detected in 8 papillary pre-neoplastic lesions and in four pRCCs. In addition to the cytoplasmic staining nuclear positivity for TXN was seen in two pRCCs. The clear cell papillary tumour was negative for both antibodies. Six cRCCs displayed cytoplasmic and two of them nuclear TXN expression as well. Two of six cRCC showed weak cytoplasmic expression of TXNIP, one of them also displayed scattered nuclear positivity. Five of the 6 cRCC displayed strong nuclear immune reaction in stromal endothelial cells. Expression TXN and TXNIP has been analysed in 691 cRCCs and 100 pRCCs obtained from the general population. None of the 691 cRCCs showed nuclear staining with TXN and TXNIP and only 3 of 100 pRCCs displayed nuclear positivity with the TXN antibody. The results of TXN and TXNIP immunoreaction in ESRD/ACRD associated tumours included in this study are summarized in Table 1.

**Table 1:** Expression and cellular location of TXN and TXNIP in ESRD/ACRD-associated tumours and tumours of the general population

Antibody		ESRD/ACRD Tumours				Other renal tumours	
		EVT (6)	chRCC (4)	cRCC (6)	pRCC (5)	cRCC* (691)	pRCC (100)
TXN	cyt	6	4	6	4	488	41
	nucl	5	2	2	2	-	3
.TXNIP	cyt	5	4	3	5	179	81
	nucl	2	1	2	-	-	-
	end-nucl	-	-	5	-	512	-

*EVT* eosinophil-vacuolated tumour, *chRCC* chromophobe RCC, *cRCC* conventional RCC, *pRCC* papillary RCC, *cyt* cytoplasm, *nucl* nucleus, *end* endothelial. *Ref. [22].

## Discussion

Kidneys with DN express significantly higher levels of TXNIP than control kidneys and increased TXNIP level is associated with excessive extracellular matrix (ECM) production [2, 18]. TXNIP deficiency protects against the development of DN and inhibition of TXNIP can reverse the ECM deposition [19]. Increased expression of TXNIP in ESRD/ACRD kidney leads to elevated level of ROS which in turn increases the inflammation and interstitial fibrosis. Macrophages in the inflammatory microenvironment (IME) of ESRD/ACRD kidneys strengthen the oxidative stress and induce the production of profibrotic cytokines such as IL6 and TGF-beta [10].

As we showed in this study, endothelial cells of arteries embedded in hyalinised inflammatory stroma display strong TXNIP expression. TXNIP promotes endothelial cell survival and angiogenesis by activating NF-kB transcription factor and play a crucial role in vascular endothelial dysfunction in DN [13, 14]. TXNIP-TXN complex regulates reactive oxygen species (ROS) generation and when ROS is elevated, it can induce long term vascular complications in DN. The strong arterial and arteriolar sclerosis causes slow or blocked blood flow in ESRD/ACRD kidneys which further increases the oxidative and metabolic stress.

Elevated TXNIP expression and oxidative stress influence several biological functions, regulates cell growth, differentiation, and apoptosis. In normal kidney cells TXNIP and TXN are expressed in the cytoplasm of distal tubular cells and stromal endothelial cells as well as in proximal tubular cells, respectively. However, TXNIP can move to the cell membrane and mitochondria leading to mitochondrial autophagy [20]. It was shown that forced expression of TXNIP in isolated microvascular endothelial cells results in its nuclear translocation and activation of the NF-kB pathway leading to expression of pro-inflammatory cytokines such as IL6 [21, 22]. In this study we found a strong nuclear expression in stromal endothelial cells in cRCC developed in ESRD/ACRD. Previously, strong TXNIP staining was seen in the nuclei of endothelial cells of tumour supporting capillary meshwork in 512 of 691 cRCC occurring the general population [17]. The overwhelming majority of 179 cRCC with TXNIP positive cytoplasm displayed higher nuclear grade. Only 3 out of 100 pRCC showed nuclear positivity with the TXN antibody. Strong expression of TXN and TXNIP in cytoplasm, at cell membrane and translocation into nuclei of EVT cells, associated precursor lesions as well as in ESRD/ACRD associated tumours suggest as the involvement of TXNIP/TXN signalling in the development of these unique type of tumours.

The impaired TXNIP/TXN redox system together with the stromal inflammation, vascular alteration leads to increased oxidative stress in ESRD/ACRD kidney. In cells exposed to oxidative stress TXN protein is imported into the nucleus by karyopherin alpha and activates several transcription factors associated with cell proliferation [23]. There is a direct association between the ERK1/2 MAP Kinases signalling pathway and TXN nuclear translocation under oxidative stress [24]. We have detected nuclear expression of TXN in EVT and their precursor lesions. Nuclear expression of TXN was seen at less frequency in other types of tumours such as chRCC, pRCC and cRCC, which occurs not only in ESRD/ACRD kidneys but in the general population as well.

One of the common mediators of carcinogenesis is the imbalance of oxidative stress induced by inflammation. Expression of IL6 and TGFβ in ESRD/ACRD kidneys can trigger the generation of reactive oxygen species (ROS) in phagocytic cells of IME [10]. The imbalance of TXNIP/TXN redox system may also increase the oxidative stress. ROS causes mitochondrial and genomic DNA damage which may also contribute to tumour development [11, 12]. Elevated levels of ROS can impair the function of important proteins, interfere with metabolic pathway, and contribute to genome damage and in this way to initiation of cancer in ESRD/ACRD kidneys. Each clinically detected tumour in ESRD/ACRD kidney is accompanied by multiplex pre-neoplastic lesions showing similar histological pattern, which may explain the field effect of imbalance of TXNIP/TXN redox system and elevated ROS [10].

## Conclusion

The frequency, increased level and cellular localisation of TXNIP/TXN in tumours of ESRD/ACRD kidneys versus tumours of the general population suggests their role in ESRD/ACRD tumorigenesis. The imbalance in TXNIP/TXN expression and stromal inflammation leading to increased level of ROS may explain the frequent development of precursor lesions and cancer in ESRD/ACRD kidney. The increased expression of TXNIP and nuclear translocation of TXN in EVT and their precursor lesions indicates possible role of impaired regulation of TXNIP/TXN redox system in development of EVT.

## Data Availability

All data obtained in this study are included in this article. The data are available from the corresponding author upon reasonable request.
